# Natrum Muriaticum

**Published:** 1898-09

**Authors:** 

**Affiliations:** Kiel


### NATRUM-MURIATICUM.

By Dr. Wassily, Kiel.

[Translated from the *Zeitschrift des Berliner Vereins Homöopathischer Aertze*, by A. McNeil, M. D., San Francisco, Cal.]

Natrum-mur. is one of the most important remedies of the homœopathic treasury, and better adapted than any other to demonstrate to our opponents the truth of Hahnemann's development of drugs by potentization. As in some regions certain forms of disease always exist, so we find in this locality that one remedy cures diseases which differ widely from each other (diagnostically, Trans.) Natrum-mur. is such a remedy in Schleswig-Holstein. Formerly much malaria manifested itself here, but now it is rare; but instead of it, types of disease occur daily which reveal a slumbering malarial influence, for which Natrum-mur. always proves itself to be the curative agent. The pathologico-anatomical images are so different that a healing artist who searches for a homœopathic remedy in the way the allopaths do will grope in darkness and obtain transient results. Only by taking the totality of the objective and subjective symptoms, and tracing out the antecedents, can the right choice of the remedy be made. One must free himself entirely from the pathologico-anatomical ideas and the names associated with them, and individualize to the finest shades, and his labor will be rewarded with success. As examples of the administering of remedies and actually curing are the best instruction, I give in the following a number of cases of disease which I have treated in the last years. The Natrum-mur. cases occur more frequently in the spring of the year.

### Case I.—Mental Depression.

Mrs. H., aged thirty-nine, consulted me January 15th, 1895, on account of the following complaints: She has suffered for years from an increased despondency, which began during a three-years' absence of her husband. She is somewhat anæmic, and in her youth was *very chlorotic*. She suffers moderately from a pressing headache. Lately, almost regularly *from 9 to 11 A. M., she has so violent* a palpitation of the heart *that she must sit down.* Mentally and physically she is intensely miserable. Sometimes such a feeling of grief overcomes her that she weeps for hours. *Every expression of sympathy is offensive to her;* she prefers to be alone then. She suffers from oppression and anxiety around the heart, and thinks much about disagreeable things that have happened to her. Menstruation regular, but profuse; suffers much the first day. Her sleep is disturbed by dreams, and she is very sleepy in the daytime. *She longs for the open air*, yet she cannot walk far. Her appetite is variable; stool and urine normal.

Natrum-mur. 200, a dose each evening for three days. In two weeks she is better, but not in all respects. Mentally freer and physically better; the palpitation has almost ceased. Continued the Natrum CC., one dose a week. (Why? Trans.) Her state of mind in the following weeks perceptibly better. She again enjoyed social pleasures, etc. In April she was attacked by influenza, attended by the most violent pains in the head and loins; Bell. and Rhus soon caused improvement; but there still remained considerable weakness, with chilliness and thirst in forenoon, and China and other remedies did not cure, and one day the former mental depression, with great anguish of mind, again returned. I gave Natrum-mur. 6, and after three days the 500th, and in a short time she was as if transformed. Even to the husband the improvement after the last medicine was so striking that he could not sufficiently admire it. I continued the medicine at long intervals, and a perfect cure resulted.

### Case II. Cephalalgia.

A slender lady, æt. forty-eight, consulted me for the following symptoms: April 28th, 1894, violent throbbing and shooting pain along the course of the trigeminus. Touch and lying down aggravate. The pains set in almost regularly every forenoon at ten o'clock. When the pains are most violent lachrymation accompanies them. At night she is free from pain. *In the forenoon there is chilliness, with thirst.*

After the ineffectual administration of Spigelia 6, which only slightly alleviated, I gave Natrum-mur. 200, three doses of five pellets each, to be taken in the evenings.

In eight days she was permanently free from pain. On the first day there was a violent aggravation.

### Case III. Disease of the Cornea.

March 15th, 1895. Miss G., æt. thirteen, has suffered for four years from constantly recurring ulcers of the cornea—both eyes. She has been twice at Sylt (a watering-place on an island of the North Sea, Trans.), which did her much good both times. There are two ulcers, the size of pin-heads, on the left cornea, near the edge. The lids are much reddened, the conjunctiva but little. She complains of burning lachrymation, and sensation of sand in the eye in the morning. Photophobia is scarcely present, only *sunlight is not well borne*. The child is somewhat chlorotic, and suffers from palpitation of the heart, especially when ascending stairs and running fast. Natrum-mur. 30, two doses a day for four days. In eight days one ulcer was healed, and after a few days more the other also. No cloudiness remained.

April 27th, 1895. She again complains of palpitation of the heart and irregular stools. Natrum-mur. 200, one dose a week for two months. After that she remained well, and the inflammation of the cornea did not return the following year, although she did not go to Sylt.

### Case IV. Disease of the Lachrymal Sac.

August 8th, 1895. Miss G., æt. thirty, suffers from a swelling of the inner canthus of the right eye, as large as a bean. When pressed, a watery secretion, which is liable to become cloudy, escapes. The conjunctiva palpebrarum reveals a moderate vascular injection. Her general health is not good. She has much *headache, usually in the forenoon, with improvement towards evening*. She has pressure in the stomach in the afternoon, *thirst*, bowels inactive. The urine frequently has a *reddish sediment;* menses regular, with profuse leucorrhœa before and

*after.* Windy and wet, cold weather aggravates the complaints of the eyes. Disposition depressed and irritable. Natrum-mur. 200, a dose night and morning for three days.

September 6th. The general condition is much better; pressure in stomach is gone, and the headache nearly so. No essential difference perceptible in the lachrymal sac. Natrum-mur. 200, a dose every week.

November 20th. The enlargement of the sac has disappeared; only with difficulty may a drop be squeezed out. Natrum-mur. 300, a dose every ninth evening.

December 21st. The patient wrote me, in speaking about the case of the child, that she has no symptoms of the disease in her eyes remaining, and her general health is perfect.

### Case V. Chronic Nasal Catarrh.

July 25th, 1896. Mrs. H., æt. forty-eight, has suffered for about two years from a chronic nasal catarrh, for which she has tried many kinds of treatment. The mucous membrane of the nose is reddened and swollen. *She has constant discharge, with loss of smell;* part of the time *it is fluent, part thick;* at times there is a profuse flow of clear water from the nose; usually this is in the *forenoon.* She also complains of weakness of the eyes, and heaviness of the lids; palpitation of the heart when ascending stairs, sometimes thirst; pappy stools; *sadness.* Natrum-mur. 12, five drops twice a day.

August 4th. Catarrh unchanged; heaviness in the lids. Natrum-mur. 200, a dose every fifth evening.

September 17th. Catarrh considerably better; the watery flow has ceased, but much sneezing has occurred. Cyclamen 30, a dose every fifth evening.

September 29th. Catarrh not much better, but certainly no worse. For a time she has fulness after eating, pressure in the stomach and dryness of the mouth. Natrum-mur. 200, a dose every week.

October 20th. I only noted, "better in every way." Prescription the same.

December 29th, 1896. The patient says she is cured; her sense of smell is restored.

### Case VI. Gastric Disorder.

April 17th, 1895. Mr. J., æt. forty-six, has had this derangement for two years, for which he has taken many medicines. *Almost every evening pains in the region of the stomach,* going then to the back, *with fulness and distension.* Acids disagree, fats still more. He often has a *feeling of hunger* in the evening, when he must eat, or the pains become intolerable. His urine is dark and strong-smelling. Palpitation of the heart is frequent. Heartburn. Sleeps badly before midnight. He is *irritable and out of humor.* He is *worse in the spring of the year.* He suffered from malaria when a child. Natrum-mur. 200, a dose every fifth evening of five pellets each till better, then one every week. In four weeks he announced considerable improvement, scarcely any pain, disposition excellent.

May 30th. He has nothing of which to complain, so that no further medication was necessary.

### Case VII. Gastric Disorder.

October 21st, 1895. Mrs. W. A., æt. fifty, has suffered for years from pains in stomach, for which she has taken many medical mixtures. These pains occur every day and at any time of the day or night. No appetite; thirst; eructations which do not relieve; palpitation of the heart when ascending stairs; cold feet; *sleepiness in the morning;* menses still continue; cannot endure sultry air, and when bad weather sets in she feels a "fever through the body." Natrum-mur. 30, a dose every fourth evening.

November 16th. She is better in some regards, only now and then she feels a slight pressure in the stomach. Natrum-mur. 30, a dose every week.

December 26th. She brought her daughter to me, who is suffering from chlorosis. She says that she herself is entirely well. The daughter was helped by Natrum-mur., as is often the case.

### CASE VIII. GASTRIC DISORDER.

February 15th, 1895. H. St., æt. thirteen, has had gastric complaints for two or three years. She has pains in the stomach when waking, and goes to bed with them. She looks very chlorotic. She is *very thirsty;* appetite very good; aversive to butter and sour *black bread;* risings of food and sour belching; frequent palpitation of the heart; stool irregular; urine on standing deposits a *red sediment;* sometimes she is very *irritable and at other times very hilarious.* Her mother was chlorotic for years, and has suffered from intermittents. Natrum-mur., a dose every fifth evening.

March 17th. Her health is very much better. The pains in the stomach have not returned since the third powder. She still has the uprisings.

Natrum-mur. 300, a dose every week.

April 26th. Her complexion is entirely changed, and her general health is perfect.

### CASE IX. RHEUMATISM OF THE JOINTS.

October 4th, 1896. Miss A. T., æt. nineteen, has suffered for five days from violent burning pains in the ankles and knees. These joints are slightly swollen. She is chlorotic, and in early childhood had a valvular disease. Appetite entirely gone; she is *thirsty;* violent *palpitation of the heart;* dry heat with red cheeks; constipation; pains day and night. Ferrum-phos. 6 in watery solution, a teaspoonful every three hours.

October 6th. Condition worse; she now has pains in the shoulders and neck, *stitches in the finger-joints and sweat in hands;* sadness. Sepia 6, eight pellets in a half-glass of water, a teaspoonful every three hours.

October 8th. But little change; palpitation aggravated when lying on the left side. Natrum-mur. 30, in watery solution, a teaspoonful every three hours.

October 10th. She is considerably better; pains only in the knees and ankles; palpitation only a little better. Medicine the same.

October 12th. Still better; appetite has returned; stool without an injection; sleeps well. Natrum-mur. 30, a dose night and morning.

October 15th. She came to my office almost free from pain. Natrum-mur. 30, a dose every week for the chlorosis, which entirely disappeared under the use of the remedy.

### Case X. Impotence.

March 3d, 1896. Mr. S. suffers from the results of onanism, the most important of which is impotence, from which he has suffered two years. During stool, which is usually hard, but at times fluid, he has *erections and discharge of prostatic juice;* he feels weak in the knees and back, and sometimes has pulsation there; he has nocturnal pollutions as often as three times a week; heart palpitates on slight occasions; *sleepy* in the daytime. Silenium 30, a dose every fourth evening.

April 24. No change. Natrum-mur. 30, in the same way.

May 21. Considerable improvement. The complaints which occurred at stool are gone. Natrum-mur. 200, a dose every week.

June 29th. The improvement has progressed. The weakness is now scarcely perceptible.

September 24th. He reported that he had accomplished coitus, although not perfectly. His general health is good. Prescription the same.

January, 1897. He reported his health as perfect in every regard, and needs no medicine.

### Case XI. Gleet.

February 27th, 1896. C. K., æt. 40, contracted gonorrhœa six months ago, for which he took weak injections of Nitrate of Silver for two weeks. The flow ceased, but after twenty-one days returned. Since then he has used all sorts of things, but in vain. There is a watery, grayish mucous discharge, particularly in the morning, and at all times by pressing on glans. *Pain when finishing micturition and afterwards;* he feels weak; has catarrh of the throat with much hawking of mucus, and a *sensation of a hair in the back part of the tongue;* constipation; sadness. Natrum-mur. 12, five drops in a teaspoonful of water, night and morning.

March 14th. The discharge is only in the morning, and obtained then only by pressing on the glans; there is a watery discharge, but not enough to form a drop. The catarrh in the throat and the general condition are considerably better. Natrum-mur. 200, one dose a week.

March 24th. There is no discharge, but he believes that at times the meatus adheres occasionally. The urinary complaints have ceased for weeks. The same prescription. I met him weeks after and he said he was entirely well.

The mental condition of the Natrum-mur. patient nearly always is depressed, particularly in chronic diseases. It may increase to the extent of a pronounced melancholy. *Aggravation from consolation is characteristic.* When most melancholy prefers solitude; when sympathy is expressed for them they either leave the room or they become irritable or in silence give free vent to their tears. Others are irritable, depressed, and incapable of performing either mental or physical labor. Dwell obstinately on past disagreeable occurrences; but sometimes in the evening cheerful, hilarious, and equal to any task; young girls at such times dislike going to bed at a reasonable hour. The mental phenomena are as changeable as the physical ones. The headaches differ much, are usually seated in the forehead, are pressing and pulsating, with aggravation from mental exertion. The pains may be very violent, the tongue is usually dry with them, adheres to the roof of the mouth, although it looks moist. School children of from ten to twelve suffer from dull headaches with sensation as if the eyes were bruised, with aggravation when moving them. It also has a headache with sharp pains and feeling as if the head would burst as with Bryonia. Or headache with partial blindness—blindness before the headache occurs with Sepia, Gelsem., Hyos., Iris, Kali-bichrom., and after the headache with Silicea. Neuralgias which rise and fall with the sun, or occur periodically, or at a definite time of day (nearly always in the morning or forenoon, Trans.).

Natrum-mur. is one of the most important remedies for impoverishment of the blood. It acts on the eyes in retinitis, which depends on a general failing of the vital powers. It acts also in amblyopia and amaurosis. The muscles of the eyes are weak and stiff on moving them. The letters run together when reading; he sees sparks, zig-zags and points before the eyes. In scrofulous opthalmias it is characteristic for a disposition to pass from one eye to the other; it is often indicated after the use of Nitrate of Silver. The ulcers are superficial, cause but little injection, and seldom leave cloudiness of the cornea. The lachrymation is usually acrid and burning, not, however, as much so as in Arsenic cases. The lids may be spasmodically closed. There is nothing characteristic in its affections of the lachrymal glands and ducts; the totality decides the choice. The protrusion of the eyeball reminds us of the beneficial effects of Natrum-mur. in morbus basedowii.

Noises in the ears, roaring and ringing, as well as hardness of hearing, are curable by this remedy.

Chronic fluent coryza is sometimes attended with dryness of the nose. In the same way the patient complains of dryness of the mouth, although the tongue may appear moist. Much mucus collects in the throat, which the patient must hawk up, particularly in the mornings. Vesicles and burning on the tongue, and a sensation as if there was a hair on it. Eruptions around the mouth and vesicles like pearls on the lips are characteristic in intermittents (also of Rhus, Trans.).

Natrum-mur. is a grand remedy for diseases of the stomach and intestines. Thirst is never really absent; the other phenomena differ extremely in individual cases. Pain or pressure in the stomach sets in either when the stomach is empty, while eating, or one or two hours after. A characteristic which is seldom observed is *sweat while eating*. Heartburn, warm symptoms, and sometimes vomiting, occur. The taste is often salt; craving for salt food often occurs. Frequently no appetite; sometimes the same patient has a morbid desire to eat—canine hunger. There is aversion to fat or the exact opposite; to bread, and sometimes to meat, coffee, or water. In many persons stitches when moving quickly or riding; in some patients the spleen, in others in the region of the liver. Cutting pains in the abdomen daily are frequent. Flatulent complaints and distension of the abdomen are not so pronounced as with Lycopodium.

Stool is delayed and difficult either from inactivity of the intestines or from hardness of the stools. In other cases the stools are either pappy or watery; after stool burning pains or stitching in the anus.

The urine often has a reddish sediment; when finishing micturition and after, more or less violent pains in the urethra are frequent; slimy, watery discharge from the urethra like that of gleet.

Weakness of the sexual organs is conspicuous. The patients suffer from pollutions, even after coition; weakness of the back as a result of onanism, also incipient spinal diseases. In the diseases of women Natrum-mur. is often useful. It is indicated in hyperesthesia of the uterus. Painful coitus in anæmic women; prolapsus of the uterus; menses irregular, usually profuse; sadness; palpitation of the heart and headache frequently precede them. Leucorrhœa is often cured by it; it is acrid, watery, or greenish; the sensation of dryness also occurs in the vagina.

Natrum-mur. patients often have pains in the back; they usually have with it a desire for a firm support, such as leaning against or lying on something hard. (Rhus and Sepia both have this; they often place the forearm between the back and the chair or the back and the bed, Trans.) Weariness in the arms or legs, but especially in the knees, is very frequent; swelling of the feet in the anæmic, and sometimes a sensation as if paralyzed in one arm.

The cough of Natrum-mur. is usually dry, and is excited by tickling in the throat or the epigastrium. Or he has mucus, bloody, insipid, or salty-tasting sputa. Stitches occur all over the chest either transiently or persistently. The cough is sometimes attended by bursting pains in the head; it occurs in paroxysms, often in the morning and in the evening after lying down, or repeatedly through the day with periods of hours between.

There is no remedy which has such different or changeable symptoms in the heart as Natrum-mur. The pulsations may be very weak or very strong, they are often felt through the entire body. Palpitation occurs while at rest or when moving, as in ascending stairs, walking quick, etc. The first sound may be either increased or divided; valvular complaints may exist. The pulse is often quick and weak, sometimes full and slow, and again very irregular, and may be intermitting, particularly when lying on the left side, with throbbing felt all through the vessels.

Natrum-mur. patients mostly suffer from weariness and sleepiness in the daytime, particularly in the forenoon; are sometimes cheerful in the evening and sleepless after first going to bed. Have many dreams—pleasant, fanciful, or anxious—nervous jerks in sleep are frequent.

The diseases on the skin which are more particularly under the curative influence of Natrum-mur. are hives, which itch, bite, and burn, and often appear in the moist air of the coast; crusty eruptions, partly purulent and partly dry, situated in the bends of the limbs (inner side), behind the ears, and on the margins of the scalp. I have also relieved and cured psoriasis with it.

In intermittents the chill is most characteristic which appears between 9 and 11 A. M. Then follows heat, which may become so violent that the patient begins to be delirious, the thirst increases with the fever; gradually profuse sweat breaks out, during which the complaints which accompany the heat again disappear.

The general characteristic symptoms are the following: Great disposition to catch cold; emaciation, particularly in children who suffer from deficient nourishment; pains and complaints appear periodically; are aggravated from 9 to 11 A. M.; in hot sultry weather, in the sunshine, in the spring and fall, when touching cold things, mental and physical exertion, from laboring with the hands; from emotions, particularly anger; from ascending stairs, from exertion of vision, from external pressure (or relief therefrom), during or after eating (or relief therefrom), on the sea coast (or relief therefrom). The patients have an extraordinary longing for fresh air, but are aggravated by remaining long in the open air. Extreme changes of the symptoms in the same person, so that at one time they are the exact opposite of another time. In my experience with no other remedy but Thuja does this occur in the same degree, and which is, like Sepia, a complementary remedy of Natrum-mur.

